# Implementation of preventive measures to prevent COVID-19: a national study of English primary schools in summer 2020

**DOI:** 10.1093/her/cyab016

**Published:** 2021-04-16

**Authors:** Neisha Sundaram, Chris Bonell, Shamez Ladhani, Sinéad M Langan, Frances Baawuah, Ifeanychukwu Okike, Shazaad Ahmad, Joanne Beckmann, Joanna Garstang, Bernadette E Brent, Andrew J Brent, Zahin Amin-Chowdhury, Felicity Aiano, James Hargreaves

**Affiliations:** 1 Department of Global Health and Development, London School of Hygiene & Tropical Medicine, London WC1H 9SH, UK; 2 Department of Public Health, Environments and Society, London School of Hygiene & Tropical Medicine, London WC1H 9SH, UK; 3 Public Health England Colindale, Immunisation and Countermeasures Division, London NW9 5EQ, UK; 4 Department of Non-communicable Disease Epidemiology, London School of Hygiene & Tropical Medicine, London WC1E 7HT, UK; 5 Derbyshire Children’s Hospital, University Hospitals of Derby and Burton NHS foundation Trust, Derby DE223NE, UK; 6 Department of Virology, Manchester Medical Microbiology Partnership, Manchester Foundation Trust, Manchester Academic Health Sciences Centre, Manchester M13 9WL, UK; 7 Specialist Children & Young People's Services, West Ham Lane Health Centre, East London NHS Foundation Trust, London E15 4PT, UK; 8 Birmingham Community Healthcare NHS Trust & University of Birmingham, Allens Croft Children’s Centre, Birmingham B14 6RP, UK; 9 Oxford University Hospitals NHS Foundation Trust, John Radcliffe Hospital, Oxford OX3 9DU, UK; 10 Nuffield Department of Medicine, University of Oxford, Oxford OX3 7BN, UK

## Abstract

We examined the feasibility of implementing preventive measures to prevent SARS-CoV-2 transmission across 105 English primary schools in summer 2020 via a survey and interviews with headteachers. High rates of implementation of most recommended measures were noted with the exception of requiring 2 m distance for students, fitting hand sanitizers in classrooms and introducing one-way systems in school corridors. Measures such as regular handwashing and stopping assemblies were considered easy to implement. Majorly challenging measures included distancing between individuals (for students: 51%, *N* = 99; for staff: 34%; *N* = 98; for parents: 26%, *N* = 100), spacing out desks (34%, *N* = 99), keeping same staff assigned to each student group (33%, *N* = 97) and staggering break times (25%, *N* = 99). Rapid implementation was facilitated by staff commitment and communication among stakeholders, but hampered by limitations with guidance received, physical environments, resources, parental adherence and balancing preventive measures with learning. Difficulties with distancing for younger children suggest that smaller bubbles with fewer distancing requirements within these may be a policy option. Schools require further financial, human resource and other support for effective implementation of preventive measures.

## Introduction

The COVID-19 pandemic has affected millions worldwide. Schools are a site of SARS-CoV-2 transmission [1] despite children being less vulnerable than adults to severe disease [2–4], and potentially less susceptible to infection [5, 6]. Data from summer 2020 suggest that within-school outbreaks were more frequent among staff members [7]. On 1 April 2020, an estimated 172 countries had implemented nation-wide school closures, affecting 84% of enrolled students [8]. Countries’ responses for reopening schools thereafter varied in timing and approach [9]. Measures included re-opening to targeted students, smaller classes, keeping students in fixed groups, use of face masks, increased hygiene, use of physical barriers, temperature checks [9–11].

In the United Kingdom, a national lockdown began on 23 March 2020 [12] when schools were closed except for vulnerable and key workers’ children. COVID-19 cases declined gradually during May 2020 [7]. Primary schools re-opened in early June initially to students in reception (age 4–5), Year 1 (age 5–6) and Year 6 (age 10–11). From 15 June, secondary schools re-opened for Years 10 and 12 students and primary schools could bring back other students.

Guidance for re-opening schools and preventive measures was provided to schools. Measures included individual- and environment-level preventive measures such as allowing a maximum of 15 students per class in primary schools, consistent groups of students and teachers not mixing with others (‘bubbles’), distancing, hand hygiene, enhanced cleaning and isolation of symptomatic individuals [13, 14]. However, little is known about the extent and feasibility of implementation of these measures across English schools. The general theory of implementation (GTI) suggests implementation occurs via processes of making sense of the intervention, cognitive participation/commitment to implementation, collective organization to coordinate enactment and reflexive monitoring informing refinement, with these supported by information, material and economic resources and supportive relationships and norms [15]. Previous research supports this, identifying leadership commitment, staff buy-in and intervention workability as key [16–18]. We undertook a national study of implementation in English primary schools in the summer term, 2020 aiming to examine how schools were implementing guidelines for COVID-19 prevention, school experiences, and facilitators and challenges involved.

## Methods

### Study setting and design

This study was nested within Public Health England’s (PHE) SARS-CoV-2 surveillance study (sKIDs) of 131 English primary schools during summer 2020 [19]. Schools for sKIDs were recruited from north London, east London, Oxford, Derby and Manchester, contacted through local PHE staff, local authorities (LAs), healthcare trusts, health protection teams and Department for Education (DfE). Primary schools with 30 or more students attending the summer half-term for four or more weeks were approached and interested schools enrolled.

Our study included a cross-sectional online survey with headteachers from 131 schools participating in sKIDs and semi-structured telephone interviews with a subset.

### Instruments

Questionnaires and interview guides content was informed by government guidelines for school reopening [13, 14]. Main topics covered in the survey were measures being implemented; perceived ease of implementation, guidance received by schools; and challenges experienced. Interviews focused on facilitators and challenges associated with implementing guidance and preventive measures, and headteachers’ experience of reopening schools.

### Participant selection

Headteachers at all schools participating in the sKIDs study were invited by e-mail to participate. They were requested to complete the survey themselves or, if unable, to assign a senior colleague to do so. All headteachers invited to complete the survey could also opt to participate in semi-structured interviews.

### Data collection

Survey and interviews were conducted in July–August 2020. Surveys were administered online. The first screen provided study information and a tick box consent procedure. Participants could skip questions and cease participation at any point. Free-text boxes allowed entry of narrative data.

Semi-structured interviews were conducted by a social scientist by telephone. Study information and consent forms were circulated to participants in advance. Written consent was obtained where feasible. Additionally, verbal consent was obtained from all participants before the interview. Interviews were audio-recorded with participants’ permission and were approximately 30 min long. No financial compensation was provided.

### Data management and analysis

Descriptive tables regarding measures being implemented, ease of implementation, demographics and other data were generated using Stata (StataCorp LLC).

Interviews were transcribed from audio-recordings, augmented with notes taken during the interview. Transcripts and notes were managed and analysed using MAXQDA 12 (VERBI GmbH). A thematic analysis [20] was carried out oriented towards addressing our research objectives. The GTI served as a sensitizing theoretical lens that guided interpretation of findings [15]. Narrative data were coded thematically using a deductive approach based on the topics covered in the interview guide and concepts included in the GTI. Thereafter, further coding identified themes inductively. Finally, sub-themes were identified within each theme to further explain and characterize each theme. Free text fields from the survey were analysed thematically alongside interview data.

### Ethical approval

This study was approved by the London School of Hygiene and Tropical Medicine research ethics committee. The sKIDs study was approved by PHE as a part of its responsibility to investigate SARS-CoV-2 infections among children in educational settings.

## Results

In total, 131 schools were contacted for the survey, and 105 responded. All 16 headteachers or designated representatives agreeing to interview were contacted and 14 interviewed. Quotes in this article come from the headteacher interviews, unless otherwise indicated. To prevent disclosure, further identifiers are not provided.

### School profiles

All schools served students aged 11 years or younger, but 8.6% (*N* = 105) also served older children. Over a third of schools served 200–400 students, while 29.5% (*N* = 105) had 401–600 students on register (Table I). The median number of teachers and teaching assistants employed was 35 (range: 9–180) among the 103 schools providing this information. A median of 21 (range: 6–90) teachers and teaching assistants attended school on a typical day over the half-term, June–July 2020.

**Table I.** Size (number of students on register) of schools participating in the survey

Number of students on register	Number of schools (*n*)	Percentage of schools (%)
0–200	6	21.9
201–400	17	32.4
401–600	19	29.5
601–800	15	7.6
Over 800	27	5.7
Missing	3	2.9
Total	105	100

### Receipt of guidance and implementation of preventive measures at school

All 105 surveyed schools reported receiving guidance on preventive measures from DfE. Forty-four per cent reported receiving information from PHE and 17% from other sources including LAs, unions, and school or academy trusts or federations. Around half (51%) found this information ‘quite useful’, 38% found it ‘very useful’, 3% found it ‘not very useful’. None found it ‘not useful at all’. Some interview participants reported relying heavily on more local guidance from LAs or school trusts or federations.

Among preventive measures for staff, handwashing was reported as easy to implement by 73%, *N* = 100 (Table II). Similarly, stopping staff meetings was relatively easy to implement, but similar numbers of schools reported some challenges. Staff not coming in to work if vulnerable or living with a vulnerable person was reported by a majority as leading to some challenges, with 11% (*N* = 100) not implementing the latter. Over 20% (*N* = 99) had not implemented a policy allowing staff who could do their jobs from home to do so. Distancing for staff was reported as the most challenging with 34% reporting major challenges and 52% reporting some challenges (*N* = 98). Face masks were not recommended at the time for staff but over 20% (*N* = 100) reported they were implementing usage.

**Table II.** Preventive measures that were implemented at schools and perceived ease of implementation from headteacher survey

	Number of schools	Not implemented	Major challenges to implement	Some challenges to implement	Easy to implement
	***N***	***n* (%)**	***n* (%)**	***n* (%)**	***n* (%)**
**Staff measures**					
Requiring regular hand cleaning for staff	100	0 (0)	2 (2)	25 (25)	73 (73)
Stopping in-person staff meetings^a^	100	7 (7)	5 (5)	44 (44)	44 (44)
Staff advised not to attend work or work from home if clinically vulnerable	100	4 (4)	5 (5)	61 (61)	30 (30)
Staff advised to work from home if they live in a household with vuln	100	11 (11)	7 (7)	56 (56)	26 (26)
Staff advised to work from home if their job can be done from home^a^	99	21 (21.2)	10 (10.1)	44 (44.4)	24 (24.2)
Requiring maintenance of 2 m distance from others for staff	98	4 (4.1)	33 (33.7)	51 (52)	10 (10.2)
Staff asked to wear face masks or face coverings while at school^b^	100	78 (78)	2 (2)	7 (7)	13 (13)
**Student measures**					
Ensuring students who have coronavirus symptoms, or have someone at home who does, stay home	96	3 (3.1)	1 (1)	41 (42.7)	51 (53.1)
Requiring regular hand cleaning for students	99	0 (0)	8 (8.08)	41 (41.4)	50 (50.5)
Ensuring students catch cough or sneezes with tissue or arm	99	0 (0)	12 (12.1)	56 (56.6)	31 (31.3)
Keeping students with the same small groups at all times each day	99	1 (1)	23 (23.2)	57 (57.6)	18 (18.2)
Ensuring that the same teacher(s) and other staff are assigned to each student group	97	1 (1)	32 (33)	50 (51.6)	14 (14.4)
Requiring maintenance of 2 m distance from others for students	99	16 (16.2)	50 (50.5)	32 (32.3)	1 (1)
Students asked to wear face masks or face coverings while at school^b^	99	92 (92.9)	2 (2)	2 (2)	3 (3)
Daily temperature checks for students^b^	100	76 (76)	3 (3)	10 (10)	11 (11)
**Classroom measures**					
Ensuring students use the same classroom throughout the day	98	0 (0)	6 (6.1)	30 (30.6)	62 (63.3)
Removing soft furnishings and toys that are hard to clean	99	0 (0)	13 (13.1)	42 (42.4)	44 (44.4)
Fitting hand sanitizers in classrooms	98	23 (23.5)	6 (6.1)	30 (30.6)	39 (39.8)
Cleaning frequently touched surfaces	99	0 (0)	17 (17.2)	45 (45.5)	37 (37.4)
Removing non-essential objects from classrooms	100	2 (2)	17 (17)	47 (47)	34 (34)
Ensuring students do not share equipment or learning materials in classrooms	97	4 (4.1)	14 (14.4)	48 (49.5)	31 (32)
Scheduling more lessons and activities outdoors	95	2 (2.1)	21 (22.1)	48 (50.5)	24 (25.3)
Maintaining space between seats and desks	99	3 (3.03)	34 (34.3)	51 (51.5)	11 (11.1)
**School measures**					
Stopping large gatherings of students, for example, assemblies	99	1 (1)	4 (4)	19 (19.2)	75 (75.8)
Fitting hand sanitizers at the school entrance	98	10 (10.2)	5 (5.1)	24 (24.5)	59 (60.2)
Stopping team sports	100	3 (3)	4 (4)	36 (36)	57 (57)
Ensuring students do not carry equipment or learning materials between home and school	98	1 (1)	8 (8.2)	39 (39.8)	50 (51)
Staggering break times for different classes	99	2 (2)	25 (25.3)	45 (45. 5)	27 (27.3)
Staggering drop-off and collection times	100	2 (2)	23 (23)	55 (55)	20 (20)
Introducing one-way systems in school corridors	98	21 (21.4)	17 (17.4)	40 (40.8)	20 (20.41)
Requiring 2 m distancing for parents dropping off or picking up children	100	2 (2)	26 (26)	54 (54)	18 (18)

a Measures that were not explicitly mentioned in government guidance;

b Measures that were noted as not recommended in government guidance.

Ensuring symptomatic students stay home (*N* = 96) and requiring regular handwashing for students (*N* = 99) were considered easy to implement by over 50% while the rest reported some challenges. Getting students to catch coughs and sneezes appropriately raised some challenges for over half the schools. Keeping students in the same small groups or bubbles raised some challenges for 58% and major challenges for 23% (*N* = 99), while keeping bubbles with the same staff-members was also challenging (some challenges: 52%; major challenges: 33%). Similar to staff, distancing among students was reported as very challenging (major challenges: 51%, some challenges: 33%, *N* = 97). Of all measures, distancing for students was most frequently reported as majorly challenging. Although face masks or temperature checks were not recommended for students at the time by DfE [13], 7 and 24 schools, respectively, reported implementing these. Policies on handwashing, and catching coughs and sneezes appropriately were implemented universally.

Classroom measures that a majority considered easy to implement were ensuring students use the same classroom throughout the day (63%, *N* = 98), removing items that are hard to clean (44%, *N* = 99) and installing hand sanitizers (40%, *N* = 98) though around a quarter of schools did not install hand sanitizers in classrooms. Cleaning frequently touched surfaces, removing non-essential items and ensuring students do not share materials were reported most frequently as raising ‘some challenges’. Similarly, spending more time outdoors and spacing out desks were reported as major challenges to implement by 22% (*N* = 95) and 34% (*N* = 99), respectively.

School-level measures of stopping student assembles, fitting sanitizers, stopping team sports and ensuring students do not carry materials between home and school were reported by a majority as easy to implement (76%, 60%, 57% and 51%, respectively). Around a 10th of schools had not installed hand sanitizers at school entrances. Staggering break and drop-off times were more challenging and reported by a quarter of the schools as majorly challenging to implement. Some challenges were reported for introducing one-way systems in schools by 41% (*N* = 98), and around a fifth had not implemented them at all. Distancing for parents was also reported as having major challenges to implement by 26% and some challenges by 54% (*N* = 100).

### Facilitators of implementing preventive measures

The following themes recurred in participants’ accounts of what factors facilitated implementation of measures and school re-opening:

#### Clear guidance

Interviewees found it useful to have received government guidance to make sense of what was required. Some believed the guidance was clear and addressed important issues in a practical manner:

It [the guidance] talked about bubbles and did what it should do, as you can’t keep children socially distanced. This was reassuring in some ways because we wondered how you would keep five- and six-year-olds two metres apart from each other.

One participant noted that the criticism it received was unwarranted:

Government guidance came under criticism but it is impossible to cater to everyone. Don’t be a pedant. If you look at it from the perspective of how can we do this, it’s clear as you like.

Some participants reported their strong commitment to the guidance, ‘You can’t go too wrong by doing that’, and referred to it as ‘The Bible’.

#### Sources of support

Schools drew on diverse material, financial and information resources for implementation. Schools that were a part of academy trusts or federations reported accessing personal protective equipment (PPE) and cleaning materials through these networks. Such schools drew on these networks to obtain guidance materials, risk assessments and other similar support. Some schools drew on financial contributions from parents to pay additional costs for cleaning supplies and protective equipment.

#### Headteacher commitment, positive attitude and knowledge of community

When describing their experiences in June 2020, all participants emphasized their commitment, aligning with the GTI concept of ‘cognitive participation’, to implementation and pride at having managed to reopen their school while implementing recommended measures within a relatively short time span. As a participant described:

It’s been a labour of love, really

One headteacher noted the experience as involving

learning as you go with a steep learning curve, which we haven’t done before.

Some headteachers emphasized the importance of school management ‘wanting to have children back at school’. A positive attitude and cognitive participation was believed to be instrumental in achieving successful outcomes for implementing recommended guidance and bringing students back to school, and was rooted in individual and collective commitment to reopening schools. Many participants felt strongly that children benefited from being in school and worked towards making this possible. Headteachers were concerned about the social and emotional well-being of students not in school, some classes only being half-full in the summer term. Some felt that the gap between disadvantaged and other students was already apparent after the March 2020 lockdown and warned of this widening. The following quotation from one head illustrates headteachers’ commitment to keeping schools open:

It is right that everyone comes in. We will make it work.

Knowledge of local communities was a key resource underpinning implementation. Participants referred to their insights into communities helping them create and adapt plans. One participant emphasized the following as a mantra for successful reopening:

Know your community well. Know your children well. Know your curriculum well

Heads emphasize the importance of their having the autonomy to make decisions based on their knowledge of community, supported by official guidance.

#### Pragmatic approach and prioritization

Local flexibility was essential to ensure implementation was workable. One participant explained how the school focused on some feasible measures more than others, for example, cleaning when distancing was not possible:

We are religious about cleaning the school but not going over the top with distancing. We are following the guidelines without being ridiculous.

Participants appreciated the bubble approach, which they considered more workable than other alternatives for distancing. A participant described their experience as follows:

I was initially a bit worried about working with bubbles, but it has worked really well. Having thought this is going to be a nightmare to implement, I think that actually children and the adults at school got used to that routine very quickly. It now works like clockwork.

#### Effective communication with staff and staff support

Relational resources also supported implementation. Communication with staff, listening and responding to concerns was considered necessary for building confidence and encouraging staff’s return to work. As two headteachers explained:

Communication has been more important than ever. Staff have appreciated the direct approach we took and knowing what was expected provided them a sense of normality.

I had several one-to-one’s about how we were going to do this and do it as safely as possible. What I think helped reassure them is just having conversations, making sure people were clear that we were very safety conscious.

Heads deployed information resources to support implementation. One participant said they had spent 4 days walking through the school site with small groups of staff ‘so everyone knew what to do and staff felt better’. Having a clear plan and implementing recommended guidelines were also considered helpful in reassuring staff, explained as follows:

Staff have been reassured by processes in place and on board with coming in to work

Most participants were highly appreciative of the efforts of their school staff. They attributed the successful implementation of measures to the dedication of their staff and were grateful for their support. One head commented:

[I am] very pleased with work of our staff which has been superb. The diligence of our staff, who have risen to the challenge, has really helped.

#### Regular communication with parents

Participants noted challenges with student attendance as parental anxiety caused fewer children returning to school than expected. As with staff, communicating and engaging with parents was necessary to ensure as many students as possible returned to school. Relational resources were employed in a variety of ways, including telephoning parents/carers of children due to return to encourage this. Some schools conducted weekly check-ins with families, and home visits to support and inform parents and children. One participant said they had conducted 38 home visits per week during this time. Some participants noted that regular communication with parents was also necessary to protect students’ mental health and set up ways for students to contact the school, if needed.

### Challenges implementing preventive measures

A number of themes emerged about challenges in implementing measures in schools.

#### Limitations of guidance content and timing

There were several recurring concerns about government guidance. The short notice period was a concern. One headteacher noted that expecting schools to reopen on the Monday after holidays, felt disrespectful and did not appreciate how schools functioned. It failed to allow sufficient time for heads to develop cognitive participation for the intervention and coordinate collective action among staff. However, most acknowledged that an unprecedented situation of a pandemic created challenges for developing timely guidance.

Most objections were instead targeted at the style of communication of guidance. One theme was the large amount of guidance that was provided and the need to navigate numerous web links to access it. Many participants found this challenging, explained as follows:

The sheer volume of guidance was overwhelming – first getting through it and then implementing it. And then the guidance changes. We were inundated and it was bordering on unmanageable. It was difficult to keep on top of the constant changes

A further problem was that some headteachers found some guidance unclear and contradictory. Some also felt it left practical questions unanswered. Three survey participants noted:

Some measures seem contradictory, such as having forward facing desks during lesson times but not during lunch.

There was not enough clarity. Could be interpreted in a number of ways. Doesn't answer any practical questions such as use of sand in EYFS^1^ etc.

Implementing the protective measures is not a challenge. Responding appropriately to the inconsistent messages regarding whether or not they should be put in place was the greatest challenge.

The fact that the guidance was frequently changed was also a concern, and many participants referred to challenges with identifying updates.

Ever changing guidance has made wider opening the most stressful situation I as Principal have ever been involved in. (Survey)

Participants described how it was difficult to keep abreast of updates when these did not indicate which aspects had been modified. One participant resorted to printing copies of the guidance to be able to compare the updated guidance with the previous version to identify changes.

Another theme was what the guidance omitted, such as developing staff confidence to return to work and implement the recommended measures. Schools as a result had to draw on their own relational and information resources to develop their own guidance. In some cases, schools with a large proportion of Black, Asian and Minority Ethnic (BAME) staff reported using other sources of information (such as that produced by the BAMEed network^2^) to address staff concerns in the absence of government guidance providing such information.

Finally, headteachers of special schools highlighted that the guidance did not include specific advice for the particular challenges they faced.

[The guidance was] not always specific to a special school setting - felt like a bit of an afterthought! (Survey)

Another headteacher commented:

Guidance came late for SEN (Special education needs) schools. As we are an SEN school with highly vulnerable students it still does not answer some questions. For example, there is still not much on children who bite and spit and require physical interventions

#### Lack of teacher input into development of guidance

Many participants perceived a lack of consultation with schools, considered this disrespectful and felt that the people responsible for writing the guidance had no understanding of life in a school, as illustrated by the following quotes:

Written by those who clearly do not understand the realities of schools and their communities. (Survey)

The guidance didn't seem to come from those who actually know what it is like to be in a classroom, particularly Early Years

However, many headteachers who criticized the initial guidance were appreciative of the guidance written for September, which was perceived as clearer, more practical to implement and addressing more of headteachers’ areas of concern. Participants said that this guidance felt like it had been written after consulting with schools more directly and involving school leaders, making it more practical and useful.

#### Balancing prevention with learning

As well as workability, integrating preventive measures with schools’ learning systems was a key issue. Many participants were concerned that social distancing and various other measures could hamper teaching. A participant noted:

Social distancing in primary schools is really difficult for the children and effective teaching requires working alongside children.

Maintaining 2- or 1-m distance was considered impossible within bubbles for younger children. Headteachers worried about the negative consequences for learning of primary school teachers distancing from students. For example, teachers’ lesser assistance with letter formation was reported as likely to affect writing skills. These challenges for student learning were described as follows:

Normally in a school like this, we are hands on, over the shoulder in contact with children’s books. But since the pandemic, [we] have not been able to touch children’s books and our marking and feedback has been stunted because of that. And children perhaps have not got the full benefit of learning and assessment that they usually would have.

The arrangement of desks and chairs in rows was not considered appropriate for primary school classrooms and was believed to interfere with effective teaching. A participant explained:

Seating children in rows does not lend itself to a primary classroom.

Some participants noted that removing items that were hard to clean from classrooms meant that students were left with insufficient learning resources, such as playdough. Similarly, not allowing students to take reading books home was considered a learning loss.

Finally, participants explained that significant time assigned to some preventive measures detracted from learning time:

Handwashing with a line of 30 children waiting for sinks takes up time, as do staggered start times.

#### Challenges associated with school physical environments

Availability of material resources also limited implementation. Insufficient space was a major concern that hindered implementation of small bubbles and social distancing. Insufficient indoor space made it difficult for some schools to follow recommendations to space out desks and remove non-essential items from classrooms, as they lacked storage space. A participant describes challenges their school faced as follows:

[Our situation] makes maintaining small bubbles impossible. We also have very limited outdoor space and are surrounded by narrow streets and terraced housing, again making social distancing at the start and end of the school day extremely unlikely. (Survey).

In one school, some classrooms could not be used as they had no natural ventilation due to the nature of the building construction, creating further space constraints. Furthermore, allocating separate toilets for specific bubbles was impossible in many schools. The head of one urban school said that the school had no green space and small playgrounds, which made it impossible to conduct more activities outdoors.

Some measures aiming to minimize mixing between bubbles, such as staggered breaks and lunchtimes, were hindered by schools only have limited canteen facilities or outdoor space. For example, the headteacher of one large school commented on their having to start serving lunch at 9:00 a.m. to be able to accommodate staggered lunchtimes for the whole school using one canteen, which made it unfeasible.

#### Insufficient financial and other resources

Economic resources could also be a limited factor. Headteachers described the additional costs incurred, in a context of already-tight budgets. They described obstacles experienced when trying to claim costs back from the government and predicted that the added financial pressures were likely to damage educational outcomes. A participant said:

The cost to our school is in excess of £30,000. We are most unlikely to get help from the government because we are not in deficit budget. The teaching and learning will be affected because of this.

The headteacher of a special school that was funded through LAs noted their ineligibility to access any government funding or assistance at all, as a major challenge. In several schools, a lack of budget meant that teachers, teaching assistants and school leaders had to do the cleaning themselves.

Isolating children with potential COVID-19 symptoms was considered a challenge without adequate PPE. Participants found it difficult to obtain PPE at the time and were unhappy that this was not more easily accessible. A survey participant explained their concern as follows:

The lack of PPE to staff in schools has been incredibly poor - very few other professions have been asked to put themselves at risk in the same was as teachers.

Headteachers of special schools noted concerns regarding the unavailability of additional PPE for their needs, where physical intervention was essential with some students.

Human resources were also critical. A recurring theme was problems ensuring sufficient staff were available and keeping staff in consistent social bubbles. Staff not in work through having to shield or illness reduced the available workforce. Some schools reported staff having to change hours and stay with the children all day with limited breaks in order to maintain consistent bubbles. This was explained by a survey participant as follows:

Main challenges are around logistical timetabling and the fact that staff had to be in only one bubble. All available staff were in a bubble - staff illness was a major issue - felt like a house of cards. Staff had to change hours and were with the children all day, often with limited break.

Other staff were too anxious to return to work. One headteacher explained the reasons for low staff turnout and attributed some anxiety experienced by staff to government messaging at the time:

We had a number of members of staff who were frightened. Some of them because of age, some of them because of circumstances, some of them because the government message said you go out and die, basically. That made them feel very anxious. And having to work with children, managing them face-to-face without knowing about infection rates and how children spread it.

#### Parental non-adherence

Parent awareness and adherence to guidance were noted as a further significant challenge. Participants complained that some parents sent sick children to school, and some tried to send their children in the next day after being sent home:

Another challenge has been the pressure that some parents have put on school to take their children even when showing symptoms…Some (fortunately very few) parents will insist that their child's cough should be ignored as they are convinced that it is not coronavirus…Even after being sent home one day with instructions about isolation, they try to send them in the next.

Headteachers also worried that some parents believed that everything was back to normal and did not take necessary precautions or behave appropriately, posing additional risks to schools. They cautioned that ‘families may not do the right thing outside school’ especially as time goes by. Heads requested further public announcements to back up school messages to parents on the need to take things seriously and stay vigilant.

## Discussion

### Summary of key findings

We undertook a national study of implementation of COVID-19 preventive measures in 105 English schools in summer 2020, using quantitative and qualitative methods. Findings suggest high rates of implementation of most recommended measures with the exception of requiring 2-m distance for students, fitting hand sanitizers in classrooms and introducing one-way systems in corridors. Measures such as regular handwashing; ensuring students use the same classroom all day; stopping assemblies and team sports, were considered easy to implement by a majority. Measures reported as majorly challenging by over a quarter of schools included distancing for staff, students and parents; keeping same staff assigned to each bubble; spacing out desks; and staggering break times.

May’s GTI [15] provided a useful framework for understanding implementation as a process of sense-making, cognitive participation and collective action supported or hindered by the workability of the guidance, the material, information and economic resources available and the availability of supportive relations and norms. Our findings indicate that cognitive participation and commitment on the part of school leaders and staff were strong. Staff collectively organized and worked hard to implement preventive measures to re-open schools safely. Limitations with the workability of government guidance, availability of economic (e.g. additional funding), information (e.g. guidance on special schools) and material (e.g. space and PPE) resources could hinder implementation. Whereas processes of implementation generally require significant time to win commitment, collectively organize and reflexively monitor, implementation of COVID-19 measures had to be achieved very rapidly. However, government guidance provided a way ahead and access to support networks alleviated some resource challenges. Schools leaders were able to develop processes and implement measures at speed, facilitated by strong staff commitment, adopting a positive and pragmatic approach, and communication with stakeholders. While implementation confirmed the workability of most measures, their integration with broader school systems, and in particular classroom learning, remained problematic.

### Limitations

The sample of schools for the survey was based on schools participating in the sKIDs study, which was a convenience sample. Schools facing the greatest difficulties that did not open promptly after the half-term or had low student numbers were not included in this study. Furthermore, 105 schools that completed this survey of the 131 schools contacted were likely more engaged with the study. Similarly, interviews were conducted with participants who opted-in. By design this likely included headteachers more motivated to discuss preventive measures implemented and potentially more engaged with infection control at their schools although it might also have included those with most complaints.

Although interviews were conducted by telephone, because topics were not too sensitive, we believe the quality of the data was unaffected. Some advantages of telephone interviews were the ability to interview participants from any part of the country and to do so in the summer term, so the interview data complemented survey data.

The sample focused on primary schools and the research was undertaken when community infection rates of SARS-CoV-2 were low [21] so the findings are not directly generalizable to secondary schools or to epidemic contexts of higher infections when schools will likely experience many more challenges relating to staff absence and the need to isolate student and staff bubbles.

### Implications for policy and research

In line with another survey of education professionals [22], our study identified problems with the timing and contents of government guidance as a challenge for school leaders. Guidance would be improved by providing more notice for implementation, keeping frequency of updates to a minimum, clearly highlighting updates and making clear which measures are a priority. Guidance should have more comprehensively addressed some issues such as developing staff confidence and provisions for BAME staff. It would have been more acceptable to school staff had it been developed in consultation with schools. Previous work on pandemic influenza preparedness and response has identified inclusiveness with stakeholders’ engagement in decision-making as a key process for building trust and effective responses [23, 24].

Social distancing was considered not only difficult to implement but unfeasible for younger students and hampering teaching and learning. To this end, the bubble approach of keeping students in small, consistent groups was appreciated in that it allowed for some mixing. From the perspective of primary school headteachers, and given the lower susceptibility of young children to SARS-CoV-2 infection [5, 6], it may be more practical to require small bubbles but ease social distancing measures within these bubbles for younger students.

A number of other measures relating to fomite transmission currently recommended in guidance were also believed to hinder learning. Some, such as minimizing items carried between home and school or removing hard-to-clean or non-essential items from classrooms, are advised based on potential risk of SARS-CoV-2 transmission via fomites. Accumulating evidence suggests transmission is predominantly through droplets and/or aerosols, and evidence for fomite transmission remains inconclusive [25, 26]. If evidence comes to suggest that fomite transmission is uncommon, it may be appropriate to review these guidelines [27]. This also has implications for schools that focused on cleaning as a visible, achievable task, given lesser control over other measures. This raises questions about strategies adopted by schools for prioritizing implementation of measures and how supported they are in making these decisions in an evidence-based manner. Furthermore, enhanced cleaning measures often took a major toll on staff, who in many cases, had to do the cleaning themselves.

Schools are deploying significant resources and there is a strong case to make additional funding, PPE and staffing available to schools to facilitate implementation of preventive measures and ensure education is not further affected. Other studies have similarly reported excess costs to schools and call for further funding [28, 29], and our findings highlight the importance of this funding being provided. Limitations with school physical environments, such as inadequate indoor space, outdoor space and ventilation, made implementation of many measures challenging. Support to schools to provide more space, such as prefabricated classrooms in school grounds or hiring of local buildings, may enable better implementation. In line with other research recommending collaboration and connectedness among school leaders to face challenges posed by the pandemic [29], further support to schools by way of formally bringing them together in groups through a community of practice may promote information sharing, problem-solving and resilience.

Special schools faced particular challenges with no guidance or equipment to deal with students with complex learning and medical and needs, including biting and spitting. Such schools resorted to developing their own plans and strategies. They were also not advised on additional PPE required, or able to procure sufficient PPE. Findings suggest extra, targeted support is needed for special schools to develop, implement and fund plans for COVID-19 measures to adequately protect staff and students.

Many other studies have raised concerns regarding student mental health, physical well-being, education and wider societal losses from school closures, including increasing educational inequalities [30–36]. The social and emotional well-being of students not attending schools, and widening gaps in learning for disadvantaged students, were concerns raised in our study too. Notwithstanding greater challenges in September during wider opening to all students, study participants were committed to the value of keeping schools open and making it work so all students could return to school.

Schools in United Kingdom (UK) opened to all students from September 2020 until the end of the year. Following a rise in COVID-19 cases, another national lockdown was announced in January 2021 [37]. Primary and secondary schools were closed once again to all but vulnerable students and children of key workers. Recently, a plan to reopen to all students on 8 March 2021 has been announced [37]. Further research, building on the work presented in this article, is currently underway in both primary and secondary schools.

## Funding

This work was supported by the Department of Health and Social Care, United Kingdom. The funder had no role in the study design, data collection, data analysis and interpretation, writing or the decision to submit this article for publication.

## Footnotes

1 EYFS: Early years foundation stage. EYFS refers to standards that schools must meet for learning, development and care of children until 5 years of age.

2
https://www.bameednetwork.com/
